# The Natriuretic Peptide System: A Single Entity, Pleiotropic Effects

**DOI:** 10.3390/ijms24119642

**Published:** 2023-06-01

**Authors:** Vittoriano Della Corte, Gaetano Pacinella, Federica Todaro, Rosaria Pecoraro, Antonino Tuttolomondo

**Affiliations:** Internal Medicine and Stroke Care Ward, Department of Health Promotion, Maternal and Infant Care, Internal Medicine and Medical Specialities (PROMISE) “G. D’Alessandro”, University of Palermo, 90127 Palermo, Italy

**Keywords:** natriuretic peptide, atrial natriuretic peptide, ANP, B-type natriuretic peptide, BNP, CNP, urodilatin, dendroaspis natriuretic peptide, guanylin, uroguanylin

## Abstract

In the modern scientific landscape, natriuretic peptides are a complex and interesting network of molecules playing pleiotropic effects on many organs and tissues, ensuring the maintenance of homeostasis mainly in the cardiovascular system and regulating the water–salt balance. The characterization of their receptors, the understanding of the molecular mechanisms through which they exert their action, and the discovery of new peptides in the last period have made it possible to increasingly feature the physiological and pathophysiological role of the members of this family, also allowing to hypothesize the possible settings for using these molecules for therapeutic purposes. This literature review traces the history of the discovery and characterization of the key players among the natriuretic peptides, the scientific trials performed to ascertain their physiological role, and the applications of this knowledge in the clinical field, leaving a glimpse of new and exciting possibilities for their use in the treatment of diseases.

## 1. Introduction

The family of natriuretic peptides (NPs) represents a group of peptides mainly characterized by the amino acid structure consisting of a peptide ring with a circular structure and by activating the same family of receptors with guanylyl cyclase activity [1,2,3]. In addition to the well-known atrial natriuretic peptide (ANP) and B-type natriuretic peptide (BNP) in the cardiovascular field, we can include other members, some of which have been known for some time, such as C-type natriuretic peptide (CNP) and urodilatin (URO), and others of more recent discovery such as dendroaspis natriuretic peptide (DNP) and guanylin peptides (GPs) [1,2,3]. NPs are represented in multiple organs and tissues, and the actions they mediate therefore involve different systems. This is the first literature review which, in addition to reporting the main characteristics of the well-known NPs, has also included other family members such as urodilatin, DNP, and guanylin peptides, as well as mentioning the most recent discoveries in this field concerning linear fragments derived from NPs and positive allosteric modulators of the NPs receptors. Moreover, unlike the previous reviews, we have tried to describe the way in which the various members of the NP family interact with each other, putting different organs and systems in communication. The purpose of this review is therefore to offer an updated and most comprehensive view on the ever-evolving picture of NPs.

## 2. Natriuretic Peptides

Each natriuretic peptide has its own molecular structure and is produced in particular organs and tissues in response to various stimuli. In this section we will individually describe each member of the NP family by focusing on these aspects. Moreover, we will also portray the characteristics of the linear ANP fragments and the positive allosteric modulators of the NP receptors.

### 2.1. Atrial Natriuretic Peptide

Atrial natriuretic peptide (ANP) was the first identified member of the NP family. It was isolated in 1983–1984 from human atrial tissue as a peptide hormone with natriuretic and diuretic activity. Chromatographic fractionation of human atria revealed the existence of three forms of ANP: α-ANP, β-ANP, and γ-ANP, with progressively increased molecular weights. The larger form, γ-ANP, was found to be the precursor of α-ANP, and β-ANP was found to consist of an antiparallel dimer of α-ANP. α-ANP (also known as ANP_99–126_, defined below as just ANP) accounted for more than 90% of the ANP immunoreactivity in the plasma, and it was concluded that this is the active circulating peptide [4]. Further on, it was demonstrated that ANP, as well as other NPs, is released in response to atrial stretch. In addition to maintaining water–salt balance and blood pressure regulation, ANP also exhibits pleiotropic effects through autocrine and paracrine mechanisms, and plays an essential role in the vascular system. The gene encoding ANP, termed NPPA, is located in the short arm of chromosome 1 and contains three exons and two introns. From its transcript, an inactive peptide composed of 151 amino acids can be formed termed preproANP, which includes a signal sequence formed by the first 25 amino acids. The tissue form of the hormone 126-amino acid proANP (also known as γ-ANP or proANP_1–126_), originates from when the 25-amino acid signal peptide is removed. Pro-ANP is stored in secretory granules of atrial cardiomyocytes [5]. In the next phase, the pro-ANP is released into the blood circulation. It is sequentially proteolytically converted into two detectable plasmatic forms, ANP and NT-proANP, by the atrial natriuretic peptide-converting enzyme (corin). ProANP is released from the atrial granules stimulated by the stretch of the atrial wall in response to the increased intravascular volume (although pressor hormones could also stimulate ANP release in this context). After secretion and cleavage, ANP enters the coronary sinus and is distributed to its target organs via circulation. ANP plays a critical role in regulating blood pressure and fluid volume by maintaining the water–salt balance and regulating diuresis, natriuresis, vasodilation, and inhibition of aldosterone synthesis and renin secretion. It has also been reported that ANP prevents vascular smooth muscle cell growth, proliferation, and vascular fibrosis, and suppresses cardiac hypertrophy and fibrosis. Normal plasma levels of ANP are relatively low (10 fmol mL^−1^), but in some pathological conditions such as congestive heart failure, circulating ANP levels are elevated from 10- to 30-fold.

In cases of severe heart failure, it is also possible to detect high plasma levels of β-ANP, which is an anti-parallel dimer of α-ANP. However, it is still not clear as to the precise mechanism that regulates its formation and secretion [6].

As mentioned, the 28 amino-acid peptide (ANP) is the most represented molecular form of circulating human ANP; it contains a ring structure with a disulfide linkage [7].

In cultures of cardiomyocytes, ANP and BNP synthesis and secretion are stimulated by several cytokines and growth factors (such as IL-18, interleukin (IL)-1β, tumor necrosis factor (TNF) α, cardiotrophin-1 (CT-1), transforming growth factor (TGF)-β, and leukemia inhibitory factor (LIF), as well as neurohumoral factors (such as Ang II, endothelin-1, and α-adrenergic agonists).

ANP exercises its physiological actions at their target cells and organs through binding to natriuretic peptide receptor-A (NPR-A). NPR-A is linked to guanylyl cyclase, meaning this bind produces the intracellular messenger cyclic guanosine monophosphate (cGMP) in the target cells. Indeed, when ANP binds to natriuretic peptide receptor-C (NPR-C), a cell surface receptor also known as the clearance receptor, it is eliminated from circulation. NPR-C lacks guanylyl cyclase activity and controls the local concentrations of NPs via constitutive receptor-mediated internalization and degradation. ANP degradation also occurs through the actions of neutral endopeptidase neprilysin (NEP), which is ubiquitously expressed in the body. NPR-C-mediated degradation has been recognized as an important mechanism responsible for ANP clearance from circulation. When NPR-C is saturated, the NPs are subsequently degraded by NEP [8]. The plasma half-life of ANP in humans is approximately 2 min, with reported values falling between 1.7 and 3.1 min. It has been reported that the lung, liver, and kidneys are the sites that are mainly involved in removing ANP from circulation. Clearance of ANP ranges from 0.5 min to 4 min, which is similar to that of other vasoactive hormones, such as vasopressin and angiotensin II [9].

### 2.2. B-Type Natriuretic Peptide

B-type natriuretic peptide (BNP) was first obtained, purified, and analyzed from pig brain extract in 1988, and was therefore called ‘brain natriuretic peptide’ [10]. A few years later, Mukoyama et al. identified much higher concentrations of this peptide in cardiac tissue [11].

The BNP gene is located on chromosome 1. From it, a peptide chain of 134 amino acids called preproBNP can be synthesized (to be precise, 26 amino acids form a signal sequence followed by the remaining 108 amino acids that form proBNP). In contrast to preproANP, which has a remarkably high rate of structural homology between the species in which it is present, for preproBNP, there is homology mainly at the amino and carboxy-terminal ends. This finding, which is anything but speculative, and without interest, explains why in the mammalian species, there are fragments of this polypeptide with different lengths: in humans and pigs, for example, BNP has a length of 32 amino acids, whereas in mice, BNP has a length of 45 amino acids.

The peptide length of 32 amino acids is formed as the result of cleavage operated on the polypeptide of 108 amino acids, and constitutes the biologically active part, while the remaining amino terminal end of 76 amino acids is biologically inactive and is termed NT-proBNP; both of these peptide chains are released from the ventricles to the peripheral bloodstream. The processing of proBNP appears to take place by an enzyme called furin, which exhibits protein convertase activity, and is mainly present in the Golgi network [12].

The mechanism by which furin activity is modulated is complex and not fully understood. The enzyme acts on proBNP by binding specific amino acid sites, marked by a consensus Arg-Xaa-(Lys/Arg)-Arg sequence [13].

However, proBNP cannot be cleaved if the polypeptide chain undergoes an O-glycosylation reaction at one of these cleavage sites, which blocks furin activity [14].

BNP storage in atrial granules with ANP is limited. Still, researchers have found high concentrations of this peptide in the cardiac ventricles, where it is not stored and released in the presence of predisposing stimuli, but can be transcribed and then assembled in response to promoting circumstances such as cardiac pressure and volume overload under the control of the transcription factor GATA4 [15].

In healthy individuals without conditions suggestive of pressure or volume overload, the plasma concentration of BNP is deficient (1 fmol mL^−1^). However, the plasma concentration increases significantly in subjects with congestive heart failure [16].

The half-life of BNP is longer than that of ANP and is approximately 20 min in humans. Unlike atrial natriuretic peptide, BNP is not initially cleaved by NEP. Instead, the first six amino acids of the amino-terminal portion are cleaved by a metalloprotease called meprin A which is present in the kidney, and which only later allows the surviving peptide chain to be attacked by NEP [17]. Of course, similar the other NPs, BNP can also be removed from circulation via the NPR-C receptor, whose mechanism of action will be described in more detail later.

Since the actions of BNP are mediated via the same ANP receptors (NPR-A), the physiological effects of BNP are similar to those of ANP and will be discussed later [18].

### 2.3. C-Type Natriuretic Peptide

C-type natriuretic peptide (CNP), the third member of the NP family, was first isolated from the porcine brain in 1990. The human gene encoding CNP, known as NPPC (gene ID 4880), is located on chromosome 2 at location 2q24-, and consists of only two exons and one intron. NPPC encodes a polypeptide of 126 amino acids, with a 23-amino acid signal sequence followed by a 103-amino acid, proCNP. ProCNP is processed into its mature form by the serin endoprotease, furin. Furin, in vitro, cleaves the 103-amino acid proCNP into a 53-amino acid carboxyl-terminal biologically active peptide termed CNP-53, which is the major active form of CNP at the tissue level. Meanwhile, the shorter 22-amino acid form (CNP-22) is prevalent in systemic circulation [1]. CNP is the most expressed NP in the brain; for example, in the hypothalamus, CNP expression is 50 times higher than both ANP and BNP [19]. However, it can also be produced by vascular endothelial cells and macrophages, meaning it is widely expressed in the endothelium, which plays a role in the local regulation of vascular tone. Recent evidence suggests that CNP play an important role in numerous tasks including vasorelaxation, endothelial cell proliferation, angiogenesis, cardiomyocyte contractility, and atherosclerosis [20,21]. Moreover, considering the wide CNP distribution in tissues and its relatively low plasma concentration, it is thought to function as an autocrine/paracrine regulator and a neuropeptide, especially in the brain, the growth plates of bones, and cardiovascular tissues, but all this will be described in more detail in later sections [22,23,24]. Natriuretic peptide receptor B (NPR-B) is the principal receptor of CNP. It is a guanylyl cyclase that catalyzes the synthesis of cGMP activated by CNP. The human NPR-B gene is located on chromosome 9p12–21 [25]. The main pathway activated by the binding of CNP to NPR-B is implicated in bone metabolism; CNP stimulates long bone growth. A third natriuretic peptide receptor, natriuretic peptide receptor-C (NPR-C), through receptor-mediated internalization and degradation, is responsible for the clearance of the NPs from circulation. Beyond clearance by NPR-C, enzymatic hydrolysis is conducted by the ecto-membrane bound zinc metallopeptidase neprilysin (NEP, or EC24.11), which rapidly removes bioactive CNP products from the tissue and plasma [26].

The clearance of CNP-22 in human plasma is very rapid. Moreover, it has the shortest half-life (2.6 min) of all the NPs [27].

### 2.4. Dendroaspis Natriuretic Peptide

Dendroaspis natriuretic peptide (DNP) is the most recently identified member of the NP family. DNP is a 38-amino acid peptide isolated from the venom of the green mamba snake (*Dendroaspis augusticeps*). Subsequently, it has been demonstrated to have structural and functional similarities to the other three NPs (ANP, BNP, and CNP), particularly in sharing the characteristic 17-amino acid disulfide ring. Similar to ANP, BNP, and CNP, it induces the formation of the second messenger, cyclic guanosine monophosphate (cGMP), through binding to its receptor. In humans, DNP has been identified in the plasma of healthy subjects and myocardial cells of patients with heart failure. Evidence suggests that DNP may be the most potent of these peptides for its natriuretic and diuretic properties, probably due to its exceptionally long C-terminus amino acid “tail”.

A commercially available polyclonal antibody to DNP has been used for radioimmunoassay plasma and tissue measurements and immunohistochemistry studies [3].

Lisy et al. investigated the bioactivity of the dendroaspis natriuretic peptide in healthy dogs and animals with pacing-induced heart failure [28,29]. According to their results, DNP induced natriuresis and diuresis, decreased cardiac-filling pressure, lowered systemic arterial pressure, suppressed renin secretion, and increased plasma and urine cGMP. They also evaluated DNP levels in normal human plasma, which revealed an average of 6 pg/mL with a range from 2 to 11 pg/mL, and elevated levels of DNP in human congestive heart failure, with a mean value of 37 pg/mL and a range from 3 to 200 pg/mL, respectively [28].

In a prospective pilot study, Vini G. Khurana et al. first examined the role of DNP in a disease affecting the central nervous system. They conducted this study to measure the levels of DNP in healthy volunteers and aneurysmal subarachnoid hemorrhage (SAH) patients, and to determine whether there is an association between alterations in serum levels of DNP and the development of diuresis, natriuresis, and hypovolemia. Venous blood samples taken from SAH patients on hospital Days 1, 3, and 7 and obtained randomly in control subjects, and serum DNP levels were found to not be significantly different. However, patients with an increase in DNP levels after SAH were found to have developed a negative fluid balance hyponatremia. Although many of the functions of the NP family have been clarified, why their regulation is perturbed in SAH is unknown. However, since the number of samples was small, further studies are needed to explain DNP’s precise role in altering the intravascular volume and electrolyte balance [30].

DNP also seems to have a potentially important role in the central nervous system. In an animal model, when administered directly into the cerebral ventricles, DNP resulted in increased diuresis [31].

However, the natural origin of DNP is not clear. The presence of DNP-like immune reactivity in cardiac tissues by immunohistochemistry and in plasma by radioimmunoassay does not represent proof of the endogenous origin of DNP, as both methods are vulnerable to the artefact and lack specificity due to possible cross-reactions with unknown proteins [3]. The gene must be identified in the green mamba snake so that appropriate probes for the cloning of the human homologue can be generated. More efforts are needed to elucidate the site of DNP synthesis and to identify the gene encoding the peptide and its biological activity. For these reasons, it is currently unclear whether DNP is an endogenous entity in humans. It could be a primitive, ancestral NP, an evolutionary precursor to ANP and BNP, or even snake BNP itself since the structure of BNP varies considerably between species [3].

### 2.5. Urodilatin

**Figure 1:**
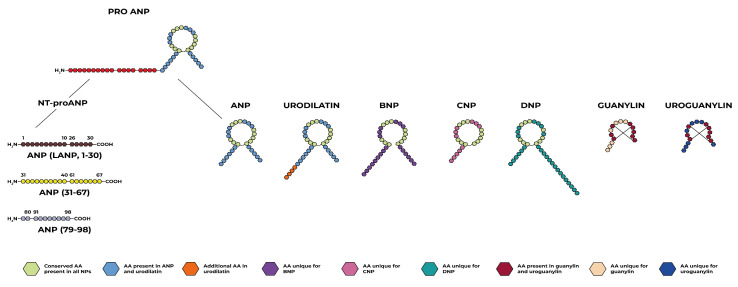
Members of the natriuretic peptide family and the connotation of similarities and differences in molecular structure.

Urodilatin (URO) is a 32-amino acid natriuretic peptide isolated from human urine in 1988 by Forssmann and coworkers [32]. It contains the amino acid sequence 95 through 126 of the cardiac proANP; is identical in structure to the circulating 28-amino acid human atrial natriuretic peptide (ANP), with the addition of four amino acids (Thr-Ala-Pro-Arg) at the NH2 terminus (Figure 1). However, contrary to all other tissues, the proANP undergoes a different post-translational process in the kidney. While in other tissues, cleavage occurs between the amino acids (a.a.) 98 and 99 to form ANP, in the kidney, cleavage occurs between a.a. 95 and 96 of the proANP, respectively [33].

While there is evidence that URO is measurable in human urine, it is not detectable in human plasma, suggesting that it is synthesized and secreted in the kidneys [34].

The existence of this biologically active substance with the ability to relax vascular smooth muscle “in vitro” was revealed by fractionating extracts of human urine by reverse-phase high-pressure liquid chromatography (HPLC) [35].

Immunohistochemical studies have localized urodilatin to the distal tubule of the kidney. URO has been deemed to be cleaved in the kidney from a prohormone identical to the proANP found in the atria [36]. URO is most likely synthesized in the distal cortical nephron and is secreted in the kidney tubules lumen where it exhibits paracrine activity. The way urodilatin interacts with and activates the receptors is similar to ANP [37]. When urodilatin is luminally secreted, it binds its natriuretic peptide type A receptor in the inner medullary collecting duct. As a result of this interaction, it increases intracellular cyclic guanosine monophosphate (cGMP) levels and stimulates renal sodium and water excretion [38].

While plasma ANP is rapidly degraded and inactivated in the kidney [39] by a kidney cortex membrane-derived metalloendoprotease, the same does not occur for urodilatin, indicating that the intrarenal ANP-receptor/guanylate cyclase system is the potential physiological target for urodilatin rather than for ANP as described in detail below [40].

The precise mechanisms that regulate urodilatin production and excretion remain to be defined. Data from Heringlake and colleagues have suggested that arterial and renal perfusion pressure changes may indeed affect urodilatin excretion. In the isolated perfused rat kidney, they found that renal perfusion and blood pressure are determinants of urodilatin excretion [41]. Furthermore, it has been reported that left atrial elongation stimulates the excretion of urodilatin [42]. In response to intravenous saline infusion and left atrial distension, Goetz and colleagues observed in conscious dogs that sodium excretion correlated better with urodilatin in the urine than with ANP in the blood.

Interestingly, this effect was abolished in heart-denervated dogs, suggesting a neuronal axis between the heart and kidney. Furthermore, using the split infusion technique, infusion of hypertonic saline into the carotid artery of conscious dogs induced an increase in urodilatin and sodium excretion, thereby suggesting a neuronal link between cephalic sodium chemoreceptors and urodilatin secretion in the kidneys [43]. However, these effects on renal function were also detectable if the kidneys were denervated [44], suggesting the presence of an additional humoral factor that transmits the increased brain sodium concentration to the kidneys. In addition, a neuronal influence was discussed, which explains an increase in urodilatin and sodium excretion after immersion in water in men. Here, the renal sympathetic nervous system or dopaminergic nerves may have led to the release of urodilatin [45]. In conclusion, several mechanisms responsible for activating urodilatin release in the kidney were discussed, such as left atrial distension, cephalic sodium concentration, renal blood perfusion pressure, and humoral or neuronal factors. However, the initial stimulus directed or transmitted through the nerves or hormones appeared to be the extracellular concentration of sodium.

Urodilatin effects are mediated by cGMP, and underlying them, there are multiple mechanisms, including vasodilation, inhibition of renal sodium reabsorption, and inhibition of the renin–angiotensin–aldosterone system [46]. All these functions will be better described in later sections.

### 2.6. Guanylin and Uroguanylin

Despite some differences from the previously described peptides, several authors include guanylin peptides (GPs) in the NP family due to their ring structure (Figure 1), guanylate cyclase-C receptor activation, and demonstrated diuretic and natriuretic effects in the kidney.

GPs are short intestinal peptides with 15–19 amino acids originating from their respective prepropeptide. They have a role in intestinal physiology but also affect the kidney, mainly on salt balance. The GP family includes guanylin (GN), first isolated from rat intestine; uroguanylin (UGN), isolated from opossum urine, and two other new members, renoguanylin and lymphoguanylin. Their actions on the intestine and kidney are well known, and it is assumed that they act similarly in several different organs that express these peptides, including the airways, pancreas, testis, salivary gland, and sweat glands. However, it is still unclear if the GP’s actions are defined through an endocrine manner, and/or if their activity is paracrine in those organs that express and secrete these peptides, such as the kidney [2].

GP production occurs mainly in the intestine; in particular, GN and UGN are produced by different intestinal mucosa cells and are then secreted into the intestinal lumen. GN is secreted by the Paneth cells, the goblet cells, the colonic Paneth-like cells, the epithelial cells, and the somatostatin-secreting D-cells of the small intestine and colonic mucosa; UGN originates from the intestinal enterochromaffin cells and intestinal epithelial cells [47]. The principal regulators of GN and UGN in the intestine are salt and intestinal pH [48]. UGN secretion is influenced by the duodenum’s and proximal jejunum’s acid pH, while GN is secreted in a neutral environment of the ileum and colorectal area. GN and UGN release are stimulated with a salt-rich diet, while their secretion is reduced with salt restriction [49]. The genes of GN and UGN are present in chromosome 1 in humans (Guca2a and Guca2b, respectively), and contain three exons and two introns [50]. Similar to ANP and BNP, GN and UGN are also synthesized as pre-pro-hormones. GN originates from its pre-pro-peptide of 115 amino acids. After cleavage, it is transformed into pro-GN composed of 94 amino acids and finally into the mature form of GN with 15 amino acids [51]. UGN is the mature form of 16 amino acids, but originates from its pre-pro-UGN of 112 amino acids and pro-UGN of 86 amino acids [52].

The activation of the guanylate cyclase-C receptor (GC-C) is the main signaling pathway in the intestine. Following binding to the GC-C receptor, GN stimulates chloride secretion through cGMP increase, thus regulating water/electrolyte transport in the gastrointestinal mucosa. These functions maintain intestinal pH and prevent hypernatremia and unwanted hypervolemic shock. Additionally, fluid balance in the intestine preserves the colonic mucus’s hydrated state, thereby influencing the growth of the commensal microorganisms and bowel clearance. Moreover, GC-C/cGMP signaling regulates intestinal barrier integrity, epithelial cell renewal, the cell cycle, DNA damage repair, inflammatory responses, epithelial-mesenchymal transition, and cancer progression. Impairment of GC-C activation causes functional gastrointestinal disorders, inflammatory bowel disease, visceral pain, and colorectal cancer [47].

In the kidneys, the GC-C/cGMP pathway seems to only be activated in the proximal tubules. Instead, a cGMP-independent signaling pathway exists in cortical collecting ducts cells. This alternative pathway passes through the activation of phospholipase A2 (PLA2), which catalyse the production of arachidonic acid, and the inhibition of luminal Kþ-channels (ROMK). It has previously been shown that natriuresis following oral salt load decreased in UGN-deficient mice. It is also known that UGN, when applied intravenously in vivo or the isolated perfused rat kidney, produced natriuresis, kaliuresis, and diuresis [2].

Assuming that the intestine is the primary site of production of GPs, these could form an endocrine axis between the intestine and the kidney. Salty meals increase GN and UGN secretion and inhibit sodium absorption, followed by anion and water secretion. Later, they are absorbed into the blood from the intestinal surface and transported to the kidney, producing hypernatremia and hypervolemia by promoting natriuresis, kaliuresis, and diuresis [47].

However, the endocrine role of GPs is still debated, as they can function as autocrine and paracrine modalities at the level of the kidney itself [2].

### 2.7. Linear ANP Fragments

In contrast to the BNP and CNP genes which each appear to synthesize only one peptide hormone within their respective prohormones (i.e., BNP and CNP), within the 126-amino acid ANP prohormone (proANP) are four peptide hormones. These peptide hormones, numbered by their amino acid sequences beginning at the N-terminal end of the proANP, consist of the ANP_99–126_ (namely α-ANP, commonly referred to simply as ANP) and three other hormones derived from the enzymatic cleavage of NT-proANP [53]. The latter are linear peptide fragments that have been identified in human blood, and consist of the ANP_l–30_, ANP_31–67_, and ANP_79–98_, respectively, which also have potent vasodilatory properties [54]. The mechanism of action of these linear fragments is similar to ANP, each activating a particular guanylate cyclase, and thereby increasing the levels of intracellular messenger cyclic GMP. For example ANP_1–30_ and ANP_31–67_ have distinct and separate receptors from the ANP receptors. Their functions have been described by several studies [55]. Respectively, ANP_1–30_ has been termed “long acting NP”, enhancing urine flow 4.3-fold while increasing sodium excretion 2.5-fold in healthy humans [53].

ANP_31–67_ defined as the “vessel dilator” is the natriuretic peptide hormone with the most significant natriuretic and diuretic effects of the NPs for the treatment of congestive heart failure (CHF). When given to patients with chronic and stable New York Heart Association (NYHA) class III CHF intravenously for 60 min, ANP_31–67_ increased urinary flow five-fold and sustained this increase for 3 h after the infusion was stopped. ANP_31–67_ was also found to have enhanced sodium excretion five-fold at the end of its infusion in subjects with CHF. Meanwhile, ANP_31–67_ caused a doubling of sodium excretion within 20 min and, 3 h after the ANP_31–67_ infusion was stopped, sodium excretion was found to still be three-fold greater than baseline sodium excretion [53].

ANP_79–98_, defined as “kaliuretic peptide” enhanced urine flow two-fold in healthy humans. ANP_79–98_ given intravenously for 60 min to NYHA class III subjects with CHF was found to have increased urine flow four-fold, and was still four-fold increased after its infusion was stopped. ANP_79–98_ enhanced sodium excretion four-fold in the first 20 min of its infusion in subjects with CHF. Sodium excretion was increased two-fold 2.5 h post-infusion. ANP_79–98_ increased the urinary excretion rate of K+ and the fractional excretion of K+ four- and two-fold, respectively. The ability of ANP_79–98_ to increase sodium excretion in individuals with CHF is of interest, as it does not increase sodium excretion in healthy individuals [53].

Since ANP_31–67_ is the natriuretic peptide hormone with the most significant natriuretic and diuretic effects, we will therefore focus our attention on this peptide.

#### ANP_31–67_

The ANP_31–67_ sequence has prolonged and remarkable diuretic effects. It performs homeostatic functions at the level of many organs and tissues, the characterization of which offers numerous possible settings for therapeutic applications. Gower et al. in 1994 demonstrated that there were distinct forms of the N-terminal and C-terminal fragments of the pro-hormone ANP in plasma, then analyzed human blood samples and subjected them to high-performance gel permeation chromatography (HP-GPC) followed by radioimmunoassay assessment, documenting that both ANP and the pro-ANP_31–67_ fragments existed and circulated as distinct entities [56]. Moreover, later studies found that plasma levels of ANP_31–67_ were at least ten times higher than those of ANP_99–126_, suggesting that the half-life of the former is longer than that of the latter [57]. The presence of the fragment in the urine being almost intact (except for two or three fewer amino acids in the terminal portion) also suggests that it resists degradation by endopeptidases (such as the neutral endopeptidase NEP), and thus explains its prolonged renal action [58]. The linear fragment mentioned above exhibits physiological effects very similar to those of the ANP ring, acting as a vasodilator, and promoting diuresis and natriuresis: both ANP and ANP_31–67_ block sodium transport at the level of collector duct cells [59]. Indeed, Vesely et al. demonstrated that the infusion of ANP_31–67_ caused a reduction in blood pressure and an Increase in both diuresis and natriuresis in healthy subjects, and also observed that the natriuretic action was more prolonged than that of ANP [55]. Another similar function between ANP and linear fragments concerns the cardioprotective metabolic effects. In fact, it is known that ANP performs cardioprotective functions thereby promoting the activation of brown adipose tissue, browning of white adipose tissue [60], and energy expenditure through mitochondrial uncoupling [61]. In the same way it is believed that linear fragments also perform cardioprotective functions on the basis of the following evidence. The linear fragments appear to play a role in exercise-related homeostasis: one interesting trial [62] documented that a single exercise session in 28 trained cyclists increased its urinary concentration considerably, no doubt in part due to the increase in venous return to the heart and the increase in heart rate linked to the single exercise session. However, another report documented higher levels of ANP_31–67_ in endurance-trained athletes compared to sedentary subjects [63], and it has been hypothesized that it plays a role in metabolism and protecting the heart, vessels, and kidneys, but this is still unknown. More specifically, the diuretic and cardioprotective effects of the linear fragment do not seem to depend on the action on arterial pressure specific to NPs: ANP_31–67_ does not activate the classic natriuretic peptide receptors such as NPR-A and NPR-B, rather it activates the cGMP pathway through different receptor structures [64]. At the kidney level, it appears to induce the formation of prostaglandin E2 (PGE2), a product of the cyclooxygenase 2 (COX-2) pathway [65], which is capable of modulating the processes of fibrosis, cell growth, and apoptosis, and acting on both inflammation and oxidative stress. Through this prostaglandin, ANP_31–67_ inhibits the sodium-potassium pump ATPase by interfering with sodium reabsorption mechanisms in collector duct cells and promoting diuresis and natriuresis. An experimental trial has also documented that intrarenal administration of the linear fragment increases creatinine clearance and inhibits renin production (in a canine animal model with unilateral nephrectomy), thereby increasing the sodium load in the macula densa [66]. In addition to the effects that have already discussed, at the renal level, ANP_31–67_, via PGE2 receptors that are G protein-coupled and designated as EP1, EP2, EP3, and EP4, respectively, promotes vasodilation and counteracts glomerular hypertrophy and the onset of proteinuria [67,68]. At the myocardial level, PGE2 receptors have various effects. EP4 is highly expressed in cardiac tissue, but the consequences must be fully elucidated. Several investigations seem to suggest that the actions of this prostaglandin, induced by the linear fragment ANP_31–67_, is protective: administration of EP4 agonists to pressure-overloaded mice revealed antifibrotic effects and limited the progression of systolic dysfunction [69]. Confirming this, Quian et al. performed an experimental model of genetically engineered animals in which they selectively inactivated the EP4 receptor at the myocardial level. In cardiac tissue, these mice did not activate the Stat-3 pathway with a sudden deterioration in systolic function after myocardial infarction [70]. However, it cannot be ruled out that EP4 exhibits its actions on cells other than cardiomyocytes, such as fibroblasts, endothelial cells, and smooth muscle cells, with a suppressing effect on fibroblast growth factors and blocking differentiation into myofibroblasts [71]. However, as it is well known, PGE2 also acts on other receptors such as EP1 and EP3, modulating downstream possible cardiac fibrosis effects through Atk regulation of cyclin D3 and p42/44 MAPK [72]. Thus, while the effects of PGE2/EP4 are believed to be cardioprotective, its action on other receptors could mediate cardiomyocyte and myofibroblast hypertrophy effects, which are the hallmarks of heart failure; it is, therefore, essential to study the molecular mechanisms induced by ANP_31–67_ in even greater detail, as understanding its role as a possible therapeutic agent requires an understanding of its effects on target cells. Regarding its role in metabolism, PGE2 acts on EP3 and EP4 receptors to regulate lipolysis and adipogenesis in white adipose tissue [73], also promoting the transformation of white fat into brown fat, thereby protecting against the risk of obesity and metabolic diseases [74]. Indeed, animal models in which mutations inactivating the EP3 receptor were induced resulted in obese and insulin-resistant phenotypes [75]. Evidence on the direct role of ANP_31–67_ in metabolic control does not yet exist. Therefore, further investigations are needed to better define the linear fragment’s impact on both metabolic pathologies and cardiovascular risk.

In the setting of heart failure, the complex role of NPs has now been well-defined as an increase in their plasma concentrations has been observed in response to the pathophysiologic cascade: volume overload, and hyperactivation of the adrenergic system and the renin–angiotensin–aldosterone system (RAAS) promotes increased levels of these NPs. However, 26% of heart failure patients do not show the expected increase in natriuretic peptide concentration either due to altered production or increased degradation by neprilysin [76]. Although it remains unclear what processing pathway NPs undergo in patients with heart failure, Winter et al., through high-pressure liquid chromatography, have noted that compared with other NPs, the linear fragment ANP_31–67_ correlates with heart failure severity [77], thus allowing for the hypothesis that beyond the role of mature NPs, the integrity of the NP system is essential for controlling heart failure, and that this assumption can be exploited for therapeutic purposes. Given its extraordinary effects at the renal level, the suitable therapeutic setting for ANP_31–67_ could be a patient presenting with heart failure and impaired renal function, or a patient presenting with cardiorenal syndrome, or to reduce symptoms in a patient with acute decompensated heart failure. Compared with mature NPs such as ANP, the effect on blood pressure and its pathophysiological consequences are less pronounced, as documented by the trial of Altara et al. [78] in a murine model with salt-induced hypertension. Undoubtedly, the road to characterizing all the molecular peculiarities of the linear fragment discussed so far is still long. Still, the potential therapeutic applicability offers exciting insights and paves the way for an increasingly in-depth and innovative research pathway.

## 3. Natriuretic Peptide Receptors

There are three receptors, well-known in their structure and mechanism of functioning, for NPs; they have a complex structure characterized by an extracellular binding domain of about 450 amino acids and a transmembrane portion of about 20 amino acids, respectively. Regarding the intracellular portion, there are differences between the three receptors: natriuretic peptide receptors (NPR) A and B display similar features, having a kinase homology domain, a dimerization domain, and a carboxy-terminal guanylyl-cyclase activity domain; they trigger the signal transmission to the intracellular district through the cyclic GMP molecule (cGMP). In contrast, natriuretic peptide receptor C (NPR-C) has an intracellular portion of only 37 amino acids and does not have guanylyl cyclase activity. Its function is to regulate natriuretic peptide concentrations through receptor-mediated peptide internalization and destruction. However, it does have other additionally known functions [79].

The interaction of NPs with their receptors constitutes the cornerstone of this complex system for regulating blood pressure and body fluid volume. However, scientific research has always recorded that the effects and functions of NPs and their receptors are pleiotropic and exert themselves on various organs and their related systems.

### 3.1. Natriuretic Peptide Receptor-A

The natriuretic peptide receptor A (NPR-A) is the main receptor for atrial natriuretic peptide (ANP), B-type natriuretic peptide (BNP), and URO; when NPs bind the receptor, a phosphorylation reaction occurs on two amino acid residues (four serine and two threonine residues) located on the N-terminal portion of the kinase homology domain, resulting in the production of the second-messenger cyclic guanosine monophosphate (cGMP) [80].

Phosphorylation of this domain is necessary and essential for receptor activation, as well as dephosphorylation, which is the mechanism for limiting receptor activity in case of prolonged exposure to NPs [81,82].

However, several studies have shown that the presence of ANP is insufficient to activate the receptor, as it appears that the presence of ATP (adenosine triphosphate) is indispensable for maximum receptor activation [83].

ATP does not represent a substrate for the phosphorylation reaction; rather, it constitutes a kind of intracellular allosteric regulator of NPR-A. Acting as a regulator, ATP exhibits a complex effect on the function of the receptor. First, it can determine a reduction in receptor activity antagonizing with ANP: ATP can limit the interaction of ANP with the receptor by reducing the number of sites available for the constitution of the receptor–ligand complex, ultimately resulting in a deactivating outcome, while diuretics such as amiloride can counteract the actions of ATP by increasing the interaction of ANP with the receptor [84,85].

The NPR-A gene and corresponding mRNA are expressed in many organs, including the heart, kidney, testes, lungs, adipose tissue, brain, and vascular smooth muscle tissue [86].

Many of the functions it performs have been understood through animal experiments: mice with deletion of alleles of the NPR-A gene showed chronic salt-resistant hypertension, together with cardiac hypertrophy and fibrosis [87]; in humans, scientists found deletion of the NPR-A gene in nine Japanese subjects, eight of whom had essential hypertension, with the ninth individual showing myocardial hypertrophy although he was normotensive [88].

#### NPR-A: Positive Allosteric Modulator

Through binding to its endogenous ligands ANP and BNP, NPR-A regulates fundamental functions in many organs and tissues. Among the most important actions are the maintenance of vascular volume and blood pressure, the regulation of heart structure, and natriuresis.

As we mentioned above, the production of the second messenger c-GMP is activated when a specific allosteric interaction occurs, mainly when ANP binds to the extracellular domain of the NPR-A and ATP binding to a putative site within its cytoplasmic region. It is believed that the binding of ATP with the intracellular kinase homology domain (KHD) has a disinhibiting action of the carboxyl-terminal guanylyl cyclase catalytic domain. This action seems to result in the production of the second messenger, c-GMP [80].

When ANP and BNP bind to NPR-A via the NPR-A/cGMP signal, in addition to regulating various biological functions, they also mediate the prevention of damage to organs and tissues. For example, the activation of this pathway protects against adverse remodeling at the cardiac, renal, and vascular levels, and inhibits the aldosterone–angiotensin–renin system. Given the favorable metabolic actions through the generation of its second messenger cGMP, NPR-A represents an important molecular therapeutic target for cardiovascular disease.

In place, therapeutic strategies targeting NPR-A include the use of recombinant synthetic peptides. In Japan and the United States, the ANP and BNP analogous, respectively carperitide and nesiritide, have been approved for treating acute cardiac failure in intravenous infusion. Other molecules that could have a better profile of action and bioavailability were also studied. In this regard, a new analogue of ANP, called MANP, is being studied to treat resistant hypertension [89,90].

Despite their therapeutic potential, the ANP and BNP analogues, administered intravenously, have a short half-life, and are rapidly degraded by neprilysin and eliminated by NPR-C. MANP has shown more excellent resistance to degradation by neprilysin; however, as a hormone, it should be administered by injection [91].

However, no molecules with a better bioavailability and resistance to degradation via NPR-C have been discovered as of yet.

Recent studies have identified positive allosteric modulators (PAMs) as possible molecules to be used for therapeutic purposes. The portion of the receptor to which the allosteric modulators bind differs from the binding site of endogenous ligands. Moreover, when positive allosteric modulators bind receptors without endogenous ligands, they do not produce any effects [92].

Recent work by S. Jeson Sangaralingham et al. identified a small molecule, MCUF-651, that acts as a NPR-A PAM, and is available for oral administration.

In this work, the authors analyzed the effects of MCUF-651 on human cardiomyocytes, human renal proximal tubular cells, and human adipocytes (visceral and subcutaneous). In particular, the authors, analyzing data on the structure of NPR-A, have hypothesized that the site to which the allosteric modulator binds is on the extracellular side [93]. The link between the positive allosteric modulators and this domain of NPR-A would seem to involve a conformational change that increases the ability of ANP to bind its receptor pocket.

The results obtained by the authors showed that the increase in the ligand–receptor bond corresponds to a direct increase in the activity of the pathway involving NPR-A and c-GMP. This suggests that the rise of the c-GMP signal pathway obtained with MCUF-651 enhances the beneficial effects on the cardiovascular and renal systems resulting from this pathway. Their data, in particular, showed that in the presence of MCUF-651, the engagement of ANP with its binding pocket occurred faster. The result of this better association was translated into higher productions of c-GMP [94].

Among the biological effects that derive from the actions of NPR-A, we remember the inhibition of the hypertrophy of human cardiomyocytes, the antiapoptotic action on renal tubular cells, and the stimulation of lipid metabolism. Based on these beneficial effects, the authors speculated that MCUF651, acting as a PAM on NPR-A, might also have a therapeutic effect by stimulating these positive biological functions. For this purpose, in an ex-vivo trial, they administered the molecule to the plasma of healthy subjects, hypertensive patients, and subjects with heart failure. The results of this trial would seem to be a good starting point for human clinical trials aimed at establishing the practical efficacy of MCUF-651 administered for therapeutic purposes. The results showed a dose-dependent increase in NPR-A activity in all patients following the administration of this small molecule, thereby confirming that MCUF-651 could be helpful in the therapeutic strategy of cardiovascular, renal, and metabolic diseases [94].

### 3.2. Natriuretic Peptide Receptor-B

Natriuretic peptide receptor B (NPR-B) constitutes the primary receptor for C-type natriuretic peptide (CNP), and has many structural similarities with NPR-A: investigations carried out on the rat receptor showed that NPR-B has 43 intra- and extracellular regions, 78% of which are identical to NPR-A as amino acid sequences [95].

The effects of NPR-B are also mediated by the production of the second messenger cGMP [95].

The affinity of this receptor for NPs is different, being higher for CNP and progressively less intense for ANP and BNP. There are two phosphorylation sites (residue four of serine and one of threonine) in the amino-terminal portion of the kinase homology domain of NPR-B through which the receptor is activated; in contrast, dephosphorylation of these residues corresponds to the shutdown of receptor activity, often as a consequence of prolonged and continuous exposure to CNP, thereby increasing the intracellular concentration of calcium [96].

The NPR-B gene and its mRNA are expressed in the heart, liver, kidney, brain, bone, uterine, fibroblasts, and vascular wall smooth muscle [97,98]. The pleiotropic effects mediated by this receptor are evident in light of experiments conducted in animal models: selective inactivation of the NPR-B gene in mice results in infertility and dwarfism [99]. Again, in mice with achondroplasia, a genetic alteration was documented in the NPR-B gene consisting of substituting a leucine residue for arginine in the guanylyl-cyclase activity domain, resulting in the inactivation of the receptor. Furthermore, in mice with defects in the endochondral ossification mechanism, aberrations were found in both gene alleles, resulting in dwarfism [100].

Moreover, in these settings, in addition to the bone alterations already mentioned, the mice had progressive and hypertension-independent cardiac hypertrophy with an increased heart rate; these elements allowed the scientists to conclude that, among the NPs, CNP has a starring role at the cardiac level, and that NPR-B (unlike NPR-A) is the receptor most implicated in the failed heart [97].

In humans, similar mutations in the NPR-B gene result in a rare form of dwarfism called acromesomelic dysplasia-type Maroteaux (AMDM) [101].

### 3.3. Natriuretic Peptide Receptor-C

The human natriuretic peptide receptor C (NPR-C) gene is located at the level of chromosome 5 (5p13–14). NPR-C has an extracellular portion structurally similar to NPR-A and NPR-B, a transmembrane domain, and an intracellular region consisting of 37 amino acids, the latter different from the intracellular domains of the two receptors described above. This receptor has no enzymatic activity, and appears to vehicle the signal inside the cell through a system involving the G protein [79]. It has three well-known glycosylation sites at the amino-terminal region; interaction with the ligand is realized by a natriuretic peptide molecule that interfaces with two receptor molecules, according to a precise stoichiometric ratio [102].

The receptor’s affinity for the NPs differs, being more pronounced for ANP, slightly less for CNP, and less strong for BNP; when receptor–ligand complex formation occurs, receptor-mediated peptide internalization occurs, and degradation takes place, respectively [103]. Internalization of NPR-C occurs even in the absence of its ligands, so this mechanism is believed to be intrinsic to the receptor and structurally predicted.

At the bone level, there is a protein called osteocrin that competes with the NPs for interaction with NPR-C (but not with NPR-A and NPR-B); therefore, although osteocrin is not structurally identical, it binds to NPR-C by increasing the concentration and bioavailability of CNP in bone tissue at specific times of development, promoting the action of natriuretic peptides in this district [104].

Certainly, among the receptors for NPs, NPR-C is the one that is most represented: it constitutes the receptor for ANP at the endothelial level, for 94% [105]. However, as seen for other natriuretic peptide receptors, NPR-C is also widely represented in different organs: the adrenal, brain, heart, kidney, intestine, and vessel smooth muscle. Again, the pleiotropic effects mediated by NPR-C were understood through experiments in animal models: hypotension, bone overgrowth, urine dilution, and reduced circulating volumes were recorded in NPR-C knockout mice [106]. Confirming the above, bone overgrowth and defects in endochondral ossification were recorded in mouse models where a loss-of-function receptor mutation was induced [107].

## 4. Physiologic Effects of Natriuretic Peptides

**Figure 2:**
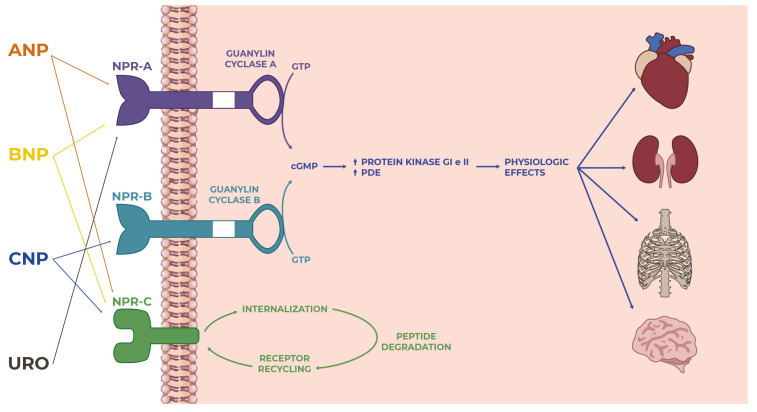
Overview of the natriuretic peptide family: from ligand–receptor interactions to intracellular signaling to actions on targets in many tissues and organs where they perform several functions.

**Figure 3:**
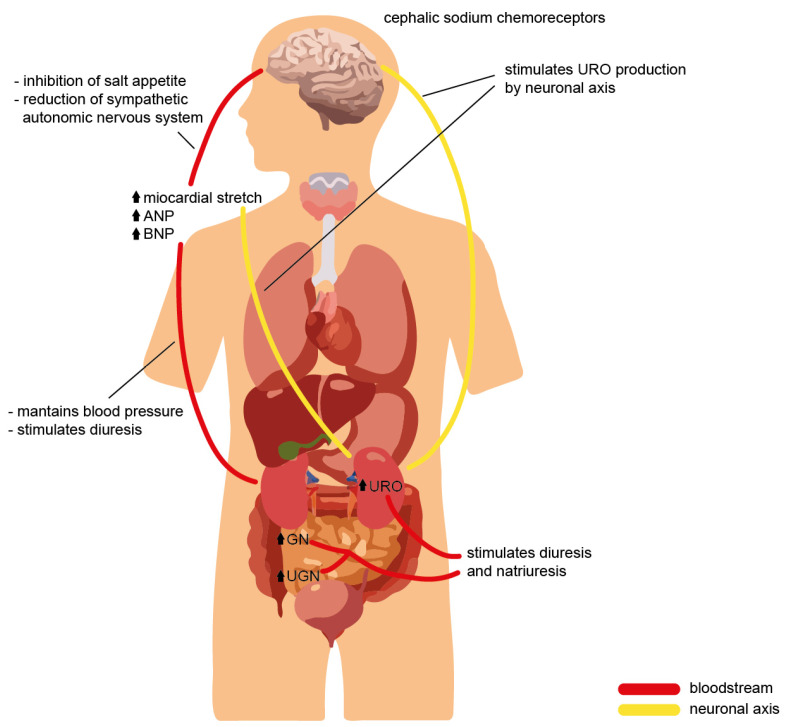
Cross-talk of the natriuretic peptide family during volume overload, with evidence of the effects on different organs and the physiological role required to ensure homeostasis.

What has been discussed so far suggests that NPs and their receptors play several physiological roles in maintaining and ensuring homeostasis in the human body by performing pleiotropic actions on multiple organs and target sites (Figure 2). The well-known effects of NPs are at the level of the cardiovascular system and bone tissue. Still, over the years, various other mechanisms have been characterized in other organs, which were made possible by the specific and individual interactions between each natriuretic peptide and the corresponding receptor in a particular site of action (Figure 3).

Knowledge of the physiological effects of NPs has been a cornerstone for modern medicine, as it has made it possible, conversely, to identify the role played by these actors in the pathogenesis of cardiovascular disease and heart failure: dysregulation of the complex natriuretic peptide machinery is crucial in the onset of the diseases described above, so much so that in laboratory diagnostics they play an ever-increasing role.

### 4.1. Natriuretic Peptide Effects on Cardiovascular System

The action of NPs on the cardiovascular system has been known for several years: the NPR-A, via activation by its endogenous ligands ANP and BNP, possesses beneficial biological properties such as blood pressure regulation, natriuresis, suppression of adverse remodeling, and inhibition of the renin–angiotensin–aldosterone system, with ANP representing the most biologically active of the two cardiac-derived peptides in activating the NPR-A [108].

The interaction of ANP with NPR-A promotes the activation of the homeostatic regulation of blood pressure, such that in laboratory mice lacking ANP or with suppression of its receptor, a 20–40 mmHg increased blood pressure was observed compared with control mice [109]. In contrast, blood pressure was found to be significantly reduced in transgenic mice with an overexpression of ANP and BNP [18].

It appears that not all of the NPs play a decisive role in blood pressure regulation: infusion of CNP results in acute reductions in blood pressure values, but in mice with a deletion of the CNP gene or the NPR-B receptor, no alterations in blood pressure regulation were reported, thus confirming that the CNP/NPR-B complex is not diriment for blood pressure control [99].

The effects on blood pressure regulation mediated by NPR-A depend on multiple mechanisms: increased urinary sodium excretion and consequent increase in diuresis, counteracting renin–angiotensin–aldosterone system (RAAS) activity, vasodilation, and increased endothelial permeability. The understanding of these processes was derived from the observation that animals undergoing atrial extract infusion incurred rapid elimination of water and sodium; in more detail, the researchers understood that the presence of NPR-A in addition to ANP was necessary to achieve the pressure-lowering effect because, in mice with deletion of the NPR-A gene, infusion of large amounts of ANP or BNP or sudden volemic expansion did not cause any natriuretic and/or diuretic responses [110]. In addition to its diuretic and natriuretic actions, NPR-A also mediates a vasodilatory process that, in acute conditions, permits the regulation of blood pressure values: infusion of ANP and BNP in laboratory animals with reduced NPR-A gene expression at the level of vessel smooth muscle does not result in the vasodilatory response that was observed under physiological conditions. However, in chronic blood pressure regulation, this process does not play such a significant role [111]. Regarding the anti-RAAS activity, Cataliotti et al. showed that in congestive heart failure patients, furosemide and BNP had favorable cardiovascular hemodynamic actions compared with furosemide alone, probably because the suppression of the RAAS may have prevented the anti-natriuretic actions of angiotensin II on the proximal tubule and of aldosterone on the distal tubule [112]. Moreover, Siragy et al. showed that in uninephrectomized conscious dogs, intrarenal ANP prevents the angiotensin II-induced decrement urinary sodium excretion and urine flow rates, thereby highlighting how ANP may play an important role in the escape from the sodium-retaining action of intrarenal angiotensin II [113].

The role of NPs in the pathogenesis of arterial hypertension has been described by interesting works. Belluardo et al. determined the levels of BNP and NT-proBNP in subjects with mild, moderate, and severe hypertension comparing them with healthy subjects. They demonstrated how in subjects with mild hypertension, BNP was unchanged while NT-proBNP was found to be significantly reduced compared with the controls. As severity increased in humans with mild-to-severe hypertension, both BNP and NT-proBNP levels were increased while not being affected by the presence of left ventricular hypertrophy. The lack of activation of BNP, together with the reduction of NT-proBNP in mild hypertension, may represent an impaired response of the BNP system in the early phase of hypertension. The later activation of both forms of BNP may be a late compensatory effect, as it correlates with the severity of hypertension rather than cardiac hypertrophy [114]. Furthermore, Macheret et al. demonstrated the existence of an impaired production and/or release of Nt-proBNP along with a concomitant reduction of BNP and NT-proBNP in the early stages of hypertension, with a significant elevation observed only in stage 2 hypertension. Moreover, they simultaneously demonstrated a lack of compensatory ANP elevation in advanced hypertension [115].

In consideration of the evidence such as those just mentioned, the efforts of the researchers are concentrated precisely on the NP system to find a new and more effective treatment of arterial hypertension. Since ANP is the most relevant component of the family that is able to modulate blood pressure and contribute to hypertension development, it is therefore expected that ANP-based therapeutic approaches may give a significant contribution to the development of efficacious therapies against hypertension. Since native ANP cannot be administered due to its short half-life, several approaches were attempted over the years to overcome the difficulties inherent to ANP instability. These approaches included ANP recombinant and fusion peptides, inhibition of ANP degradation by neprilysin inhibition, gene therapy, and designer peptides. For example, Chen et al. showed the BP-lowering properties of a novel ANP-based compound (MANP [mutant ANP]) in humans. MANP is an ANP mimetic retaining the 28 amino acids of ANP but including a novel 12 amino acid extension to the carboxy-terminal end. The latter confers a greater resistance to enzymatic degradation by both NEP (neprilysin) and IDE (insulin-degrading enzyme) and reduces removal by NP clearance receptors. The study by Chen et al. demonstrated the natriuretic and aldosterone suppression properties of MANP, as well as a good degree of tolerability, and a long lasting and dose-dependent BP-lowering effect, with no relevant side effects during the 24 h of observation [116].

Speaking of endothelial permeability, the ANP/NPR-A system plays an essential function with a direct impact on the regulation of circulating blood volume: in murine models, the decreased endothelial expression of the NPR-A gene induced by genetic engineering techniques, led to hypertension, increased plasma volume, and reduced clearance of albumin from the vascular tree [117].

In the cardiovascular system, NPs also have the role of regulating myocardial hypertrophy and cardiac remodeling by inhibiting them; on this point, it is essential to specify that hypertrophy and remodeling are not the immediate and direct responses of exposure to high blood pressure, rather they constitute the results of specific alterations in the molecular pathways of which the NPs are vital players.

The ANP/NPR-A system regulates myocardial hypertrophy, whereas both ANP/BNP/NPR-A and CNP/NPR-B contribute to cardiac remodeling. Although long-standing hypertension can promote the onset of myocardial hypertrophy, as mentioned above, the latter is mainly dependent on the ANP/NPR-A system. In mice deficient for NPR-A, hypertrophy is much more pronounced than in the controls with regular receptor gene expression, suggesting that the absence of NPR-A plays a role in promoting the processes that trigger it. Confirming the above, NPR-A knockout mice underwent cardiac enlargement even when treated with antihypertensive drugs from birth [118]. Again, mice with reduced expression of NPR-A at the level of cardiomyocytes developed moderate myocardial hypertrophy even though they were moderately hypotensive [119].

The ANP/NPR-A interaction oversees the control of myocardial hypertrophy as the scientific literature has extensively documented; the BNP/NPR-A interaction regulates the mechanism of fibrosis: deletion of the BNP gene results in normal pressure values and regular cardiac volumes in mice, but can also generate ventricular fibrosis especially if the animal has received pressure overload [120].

In NPR-A knockout mice, echocardiographic and histologic findings showed hypertrophy and interstitial fibrosis; these effects, however, were more attenuated in mice with a simultaneous deletion of the gene of angiotensin II receptor 1A (AT1A receptor), or in which an AT1a receptor antagonist was administered. In mice with an exclusive deletion of NPR-A, administration of antihypertensives such as 6-hydroxydopamine resulted in blood pressure values that were comparable to those of mice with a blockade of the AT1A receptor (through either gene deletion or pharmacological inhibition), but did not result in any effects relating to limiting fibrosis and myocardial hypertrophy as NPR-A, as mentioned above, directly regulates hypertrophy and fibrosis independently of blood pressure [121]. These data show that the response of the cardiomyocytes of NPR-A knockout mice to angiotensin II is enhanced, and results in hypertrophy and interstitial fibrosis, again confirming the role of NPR-A as an inhibitor of the pathological processes of myocardial hypertrophy and cardiac interstitial fibrosis.

More specifically, to establish the role of NPR-A in the mechanisms of fibrosis and hypertrophy, scientists have previously crossed transgenic mice that overexpressed NPR-A with NPR-A knockout mice, obtaining mice that expressed NPR-A in the heart: these animals had similar blood pressure and heart rates to the NPR-A knockout mice, but had smaller cardiomyocytes; consequently, the levels of ANP and its mRNA at the cardiac and systemic levels were lower due to the expression of NPR-A than in the NPR-A knockout mice [122]. Conversely, in mice with a selective deletion of the cardiomyocyte NPR-A gene, serum ANP levels were found to be higher than in the wild-type mice. Moreover, blood pressure was 7–10 mmHg lower due to the NPs’ effects on both renal and vascular districts. Still, these animals’ cardiomyocyte response to pressure overload resulted in significant hypertrophy and systolic dysfunction [123].

In addition to cardiomyocytes, in the heart, NPs act on fibroblasts: the activation of NPR-A and NPR-B causes an increase in cGMP production, limits the Ang II-induced increase in preproendothelin-1 expression, and restricts the proliferation of fibroblasts themselves; by regulating the growth and proliferation of these cells, it is clear how the natriuretic peptide system governs the cardiac fibrosis pathway [124].

Additionally, the relationship between ANP and cardiac mass previously observed in animal models has also confirmed in humans: reduced circulating levels of ANP are associated with more pronounced cardiac hypertrophy in subjects with essential hypertension. Indeed, in subjects with an allelic variant of the ANP gene promoter in which the serum levels of this natriuretic peptide were reduced, hypertrophy and cardiac mass were found to be at greater levels [125]. Furthermore, in diseases such as obesity and metabolic syndrome, ANP levels are lower than in healthy individuals, probably due to both increased clearance and reduced synthesis; based on the above, an inverse–proportionality relationship between ANP levels and cardiac mass has been reported in these subjects, with pronounced myocardial hypertrophy [126]. Interestingly Cataliotti et al. showed that in patients with end-stage renal disease, elevation of the plasma BNP concentration is more specifically related to left ventricular hypertrophy compared with the other NP levels independent of congestive heart failure [127]. Similarly, Zoccali et al. found that in dialysis patients, BNP and ANP were the strongest independent correlates of the left ventricular mass index, independent predictors of ejection fraction, and predicted overall and cardiovascular mortality [128]. Based on this evidence, Cataliotti et al. used the myocardium-tropic adeno-associated virus serotype 9 (AAV9) vector to achieve continuously enhanced cardiac rat proBNP expression. In spontaneously hypertensive rats, a single systemic administration of the AAV9 vector allowed long-term cardiac BNP overexpression, resulting in reductions in both systolic and diastolic BP for 9 months after injection, proving that the long-term BNP gene delivery prevented the development of hypertensive heart disease in this population of rats [129].

In conclusion, activation of NPR-A by ANP blocks myocardial hypertrophy, whereas activation of the receptor by BNP inhibits fibrosis.

The CNP/NPR-B complex, on the other hand, appears to play a role in cardiac remodeling. In mice with induced cardiac ischemia, CNP infusion blocked postischemic remodeling, thereby inhibiting the cascade of events that drive cardiac enlargement and the onset of congestive heart failure [130].

Furthermore, in CNP knockout mice subjected to pressure overload by abdominal aortic constriction, a series of detrimental cardiac changes were observed, such as LV dilatation, a reduction in ejection fraction, and increased hypertrophy and fibrosis. The same structural and functional alterations resulted in NPR-C knockout mice, suggesting that this receptor is actively implicated in the beneficial effects mediated by CNP. Moreover, a recent study suggested the role of CNP/NPR-C pathway enhancements in improving HF [131].

Concerning vascular effects, in vitro studies had previously documented that ANP can promote angiogenesis and endothelial cell proliferation [132].

ANP binding to NPR-A is a key signaling pathway regulating normal homeostatic blood pressure. This was previously demonstrated in mice lacking ANP or its receptor NPR-A, which were found to have blood pressures that were elevated 20–40 mmHg, compared to control mice [133]. The link between NPR-A and blood pressure in mice is particularly strong, as Smithies and colleagues demonstrated that NPR-A copy number is inversely related to blood pressure in a remarkably linear manner [134]. Conversely, blood pressures in transgenic mice overexpressing ANP or BNP were substantially decreased [18]. Although the infusion of supraphysiological levels of CNP into animals acutely reduced blood pressure [135], mice lacking functional CNP or NPR-B are normotensive [99], suggesting that the CNP/NPR-B pathway is not a fundamental regulator of basal blood pressure in mice. NPR-A-dependent decreases in blood pressure are achieved through natriuresis and diuresis, vasorelaxation, increased endothelium permeability, and antagonism of the renin–angiotensin system [1].

It should indeed be noted that many of the effects described so far can be traced back to in vitro trials, and that the reality of the human body is quite different and much more articulated; the insights into the role of NPs are significant, but more information needs to be gained from in vivo trials to understand what occurs under physiological conditions and, even more interestingly, in the pathological conditions on each target district.

### 4.2. Natriuretic Peptide Effects on the Nervous System

While NPs were initially discovered in cardiac myocytes, the extensive distribution of NPs and their receptors in the brain of animal species have been repeatedly reported. NPs and their receptors were shown to be present across various neuronal structures, glial cells, and cerebral vessels. Notably, findings from animal studies suggest that NP may regulate neuroplasticity, blood-brain barrier integrity, neuro-inflammation, and memory function [136].

The complete understanding of the effects of NPs certainly also comes from assessing the distribution of receptors in different areas of the brain and the specific functions of these receptors: NPR-C is well represented in all areas of the central nervous system since its action here is mainly to block glial cell growth and proliferation [137].

NPR-A, on the other hand, is mainly located in the areas close to the third ventricle of the brain, which are not isolated from the peripheral circulation by the blood-brain barrier: this allows the receptors to bind to NPs that were produced in the central nervous system and those produced peripherally; this receptor, unlike the previous one, primarily mediates the sense of water drinking and salt appetite, with direct and preferential effects on blood pressure [138].

NPR-B is distributed mainly in the hypothalamus and other rostral regions of the brain, with regulatory effects on the sympathetic nervous system and vegetative functions.

Therefore, we have observed how NPs are produced and act at the level of the CNS. As far as the circulating NPs are concerned, although they cannot cross the blood-brain barrier (BBB), they exert their action on selected sites in the central nervous system by reaching targets outside the BBB: the median eminence of the hypothalamus or the area postrema. The NPs ANP, BNP, and CNP are produced in the brain: biologically active substances that regulate blood pressure and vasoactive amines such as endothelin, noradrenaline, and vasopressin (not angiotensin II), which induce the production of these molecules in hypothalamic neuron cultures [139].

The effects of NPs at the brain level are complementary to those on other targets, with a reinforcing impact on the latter: the inhibition of salt appetite and stimulation of the sense of thirst stimulate natriuretic and diuretic actions at the kidney level [140]. In addition, other reports show that NPs exhibit a central effect by limiting the production and release of vasopressin with direct actions on the hypothalamic nuclei and, in other cases slowing down ACTH production by acting on the pituitary gland, and thus modulating all the regulatory effects of blood pressure and circulating blood flow downstream [141].

Even more interestingly, NPs regulate the tone of the sympathetic autonomic nervous system in a negative direction, thus promoting a reduction in blood pressure. Moreover, in mouse models with genetic forms of essential hypertension, blocking the action of NPs on the nucleus of the solitary tract further increases the pressure values, thus suggesting a hypotensive effect at this site [142].

All of the studies cited so far have been conducted on animals. However, there is also evidence available showing the involvement of NPs in the human brain. For example, van der Weerd et al. showed that both NP and NPR genes are ubiquitously expressed throughout the brain in healthy humans. Furthermore, NP and NPR are present in various cellular structures, including neurons, astrocyte-like networks, and cerebral vessels. The abundance of NP and NPR in human brains suggests NP involvement across multiple brain functions. Moreover, human evidence indicates that higher plasma levels of NPs are associated with dementia and accelerated cognitive decline. Although this link was mainly attributed to cardiovascular pathologies, recent findings suggest plasma NPs are associated with cognitive decline independent of cardiovascular disease [136].

A neuroprotective effect of NPs seems to be related to cGMP. As we have also seen, NPRs are guanylate cyclase receptors, and numerous pieces of evidence show how cGMP, regardless of its source, plays a vital role in brain physiology. Its actions are significant regarding modulations of long-term synaptic activity changes in the hippocampus, amygdala, cerebellum (by pre-synaptic transmitter release), and post-synaptic functions via activation of different protein kinase G (PKG) isoforms. Recent studies have demonstrated a neuroprotective role of NPs and the cGMP signaling pathway. An increase in intracellular cGMP concentration protects neurons against excitotoxic, metabolic, as well as oxidative damage and N-methyl d-aspartate (NMDA)-induced neurotoxicity [19].

All this evidence suggests a possible role of NPs in the function and metabolism of nervous tissue and as a possible link between the kidneys, cardiovascular, and nervous systems. Further studies will be needed to clarify all these aspects.

### 4.3. Natriuretic Peptide Effects on Renal System

The natriuretic effect of NPs occurs through their flow-regulating actions at the renal level and through a direct tubular effect. The mechanism of natriuresis, once triggered, lasts longer than changes in renal hemodynamics. This is because the two effects are distinct and differentially regulated.

NPs promote the contraction of the efferent arteriole and vasodilation of the afferent arteriole, thereby generating increased blood flow in glomerular capillaries and consequently increased glomerular filtration [143]. Moreover, at the mesangial level, activation of NPs receptors promotes, through increased cGMP concentration, relaxation of mesangial cells with an increased available filtration surface area [144].

Several studies have shown that plasma concentrations of NPs not capable of increasing the glomerular filtration rate are, however, able to bring about a natriuretic effect through direct actions at the level of the renal tubules: urodilatin is an example of a natriuretic peptide produced at this site that acts by a paracrine mechanism, complementary to the systemic effects of the other NPs that have already described [32].

The complexity of the renal action of NPs is mainly due to the variety of targets at which they act: at the level of the proximal convoluted tubule NPs block angiotensin II-dependent sodium and water transport [145], at cortical collecting ducts inhibit the vasopressin-dependent transit of water [146], and at the level of the medullary collecting ducts inhibit sodium reabsorption through increased cGMP production [147].

Practical applications of this knowledge have made it possible to observe that the infusion of ANP and BNP in humans at doses slightly higher than the physiological plasma concentrations of these NPs causes increases in both diuresis and natriuresis independently of changes in blood pressure. Moreover, in the trials in which it was assessed, the result was a reduction in renin concentration and angiotensin II-mediated aldosterone secretion [148].

CNP similarly reduces aldosterone secretion, but its action on blood pressure and water and sodium excretion is lower than with other NPs [27].

We have already seen how CNP is present in both the genitourinary and renal tracts [24].

Particularly, the presence of both the peptide and its receptor in human kidneys suggests a role for CNP as a renal autocrine and/or paracrine factor. To date, however, the pathophysiological role of CNP in the human kidney remain unclear. In anesthetized rats and normal humans, CNP has been shown to be mildly diuretic and natriuretic, although other reports have demonstrated that intrarenal arterial administration of CNP has no effect on the urine volume or urine sodium excretion [149]. With this in mind, Cataliotti et al. assessed plasma CNP concentrations and urinary CNP excretion in patients with nephrotic syndrome. First of all, they confirmed the production of CNP in normal human kidneys and its localization by both in situ hybridization and immunohistochemistry [149].

Moreover, they found increased plasma CNP and urinary CNP excretion in nephrotic syndrome, the latter being significantly reduced by the low-protein diet, whereas plasma CNP remained unchanged. These findings demonstrate that CNP metabolism is altered in nephrotic syndrome, and supports the hypothesis that the increase in intrarenal release and production of CNP can be partially offset by a restriction in protein intake [149].

Despite the demonstrated diuretic and natriuretic effects of ANP and BNP, the first indications that these NPs may not be the physiological regulators of Na + excretion arise from a study in which the left atrial pressure was elevated in two groups of dogs, one normal and the other with denervated hearts. Elevated atrial pressure increased circulating ANP in similar amounts in each group of dogs. However, only normal dogs developed increased diuresis and natriuresis during these experiments [150], indicating that the renal response induced by atrial distension required intact cardiac nerves and not just the release of ANP. A study evaluating the circadian rhythm of ANP in plasma found no relationship between blood sodium and circulating ANP levels [151].

Urodilatin, the only natriuretic peptide produced in the kidney, induces the diuretic and natriuretic effects at lower doses than those required for the other NPs, most likely due to a greater resistance to the activity of endogenous endopeptidases; this feature could make the use of this molecule more convenient than the other NPs for therapeutic purposes in clinical practice, beyond only trials conducted for scientific research [152].

After isolating the urodilatin natriuretic peptide from human urine, more data have been generated supporting the view that neither ANP or BNP, but urodilatin appears to be the natriuretic peptide responsible for renal manipulation of sodium. Drummer and colleagues found it interesting that not ANP in plasma but urodilatin in urine was found to be correlated closely with natriuretic responses observed following acute saline infusion. The same group demonstrated that natriuresis following the ingestion of meals with different salt concentrations was accompanied by an increase in urodilatin excretion rather than an increase in circulating ANP [153]. In addition, a negative correlation was observed between plasma ANP and renal sodium excretion during left atrial distension in dogs with cardiac denervation [42]. This body of evidence suggests that it is urodilatin, rather than the other NPs that is primarily involved in the physiological regulation of renal sodium excretion. On the contrary, due to the rapid secretion of ANP and BNP as a response to several cardiovascular stimuli, and due to the numerous effects on the cardiovascular system, it seems reasonable to postulate that the primary target of ANP and BNP is the cardiovascular system and not the kidneys.

The importance of renal effects, and possible therapeutic implications related to the use of NPs, emerged from a trial that evaluated the effects in animal models, associated with the administration of a natriuretic peptide antagonist on A and B receptors termed HS-142-1 [154]: in both healthy animals and animals in which heart failure was experimentally induced, the drug was able to inhibit diuresis and natriuretic peptide-mediated natriuresis, elevate vascular resistance in the renal circulation, and increase the concentration of renin, aldosterone, and catecholamines [155]. In addition, in cirrhotic rats with ascites and diabetic rats, this drug caused a reduction in glomerular filtration by reducing blood flow to the kidney: this suggests that disruption of the natriuretic peptide system may play a role in the onset of these diseases [156].

Finally, as we have already reported, GN and UGN could represent an endocrine axis between the intestine and kidney, stimulating natriuresis and diuresis in response to a salty meal.

### 4.4. Natriuretic Peptide Effects on Musculoskeletal System

NPs also have a function on bone growth, long thought to be minor but now increasingly better characterized: experiments carried out on animal models using transgenic or knockout mice have made it possible to describe the role of NPs, their receptors, and the intracellular molecular signaling pathways they trigger. The precondition for understanding the role played by NPs on bone growth is a brief description of the mechanisms of embryogenesis and the development of bone segments.

In vertebrates, bone genesis occurs through two mechanisms. The first is that of “intramembranous ossification” (which, for example, concerns the flat bones of the skull) in which mesenchymal cells undergo a process of differentiation into osteoblasts that begin to produce bone matrix from ossification centers; there is no strong evidence from studies to date that suggest NPs play an active role in this mechanism specifically. The other mechanism is “endochondral ossification” and concerns the genesis of the long bones of the skeleton and vertebrae. In this process, mesenchymal cells group to form the primordial bone, at the center of which some of these cells differentiate into chondrocytes, which, through specific molecular regulatory pathways, undergo proliferation, hypertrophy, calcification, and finally apoptosis to be ultimately replaced by bone marrow [157].

Surrounding this central zone, mesenchymal cells differentiate into perichondrium cells, becoming osteoblasts and replacing part of the cartilage with bone tissue. In the future development and homeostasis of the bone in question, a key role is played by the chondrocytes, also derived from the differentiation of mesenchymal cells: as the bone develops, the chondrocytes in the central zone differentiate into hypertrophic chondrocytes, and the hypertrophic chondrocytes that have aged further in the periphery contribute to the mineralization of the bone by undergoing apoptosis; therefore, the maintenance of bone is ensured by the dynamism of these cell populations [157].

In the regulation of these mechanisms, several molecules play a crucial role, including Indian hedgehog (IHH), parathyroid hormone-related peptide (PTHrP), and members of the bone morphogenetic protein (BMP) family. Chondrocytes produce IHH and promote these cells’ differentiation towards later maturational stages, while PTHrP is expressed in the joint perichondrium and acts on proliferating and prehypertrophic chondrocytes [158,159].

Through genetic engineering techniques, transgenic or knockout mice were constituted in various animal models to study the role of each of these intracellular signaling pathways. It was found that interference with several of these molecular pathways results in profound and significant alterations in bone growth and development [160,161]

The role of NPs in bone growth and development began to be understood in 1988 when ANP was seen to inhibit DNA synthesis in epiphyseal plate cell cultures in avian tibiae, including through inhibiting the PTHrP-mediated pathway [162].

Later, Hagiwara et al. demonstrated that in mouse chondrocyte cultures, NPR-B was expressed, and that its most characteristic ligand (CNP) was found to significantly inhibit DNA synthesis [163]. Despite what in vitro studies have shown, namely that NPs acting on their receptors limited bone growth, transgenic mouse models that overexpressed BNP at the liver revealed disproportionate bone growth [164]. The researchers, in this case, hypothesized that BNP acts on NPR-B by promoting bone growth, but less intensively than CNP, which in vivo stimulates and promotes bone growth and skeletal development.

Further evidence for the role of stimulation on bone growth in vivo was derived from animal models in which a mutation had been chemically induced in the NPR-C gene or animals with a deletion of this gene, in which skeletal overgrowth was observed [106,107]. As the main role of this receptor consists of cleaving NPs, the defects in NPR-C function observed in these animals caused an excess of NPs in bone and accounted for their excessive growth.

In CNP knockout mice who developed a form of dwarfism in which chondrocytes of the proliferative and hypertrophic zone were less represented, inducing the transgenic expression of CNP at the chondrocyte level corrected this bone growth defect, indicating a paracrine action of CNP at the bone tissue level as well [165].

Selective deletions of ANP, BNP, and NPR-A genes do not result in skeletal defects, proving that the CNP/NPR-B system plays a crucial role in bone growth and homeostasis.

Another critical protagonist in this context is musclin, also termed osteocrin (OSTN), as it was initially identified as a secretory peptide from muscle and bone [166]. The preprocessed mature form of mouse OSTN consists of 130 amino acids. Its carboxy terminus contains tandem NP-like sequences separated by polybasic amino acids, presumably cleaved by peptidases. Therefore, musclin can be considered a member of the NP family without the “ring” that is the common feature of ANP, BNP, and CNP, and is mainly produced by skeletal muscles and osteoblasts [167].

In skeletal muscle, musclin improves insulin-dependent glucose metabolism and enhances physical endurance by promoting mitochondrial biogenesis through NP-induced cGMP production due to NPR-C blockade [168]. The recent significant discovery made was that this peptide binds NPR-C competitively and efficaciously inhibits NPR-C-mediated NP degradation, thereby increasing NP levels, with a consequent reduction in BP and enhanced protective activities in many tissues, including the heart. Musclin was found to attenuate cardiac dysfunction and myocardial fibrosis by augmenting the CNP/NPR-B-stimulated cross-talk of cGMP and cAMP in cardiomyocytes, and by inhibiting p38 MAP kinase (MAPK) signaling through the activation of PKG in cardiac fibroblasts. Furthermore, musclin mRNA levels in skeletal muscle were increased by physical activity and, on the contrary, were markedly downregulated in biopsies from patients suffering from HF with sarcopenia or cachexia [169]. This evidence suggests that musclin could act as a possible bridge between sarcopenia and cachexia, which are highly prevalent in advanced states of HF [170], as well as the progression of HF itself. Furthermore, it could represent the biological basis of the vicious circle between reduced physical activity, decreased muscle mass, and HF in older patients [131].

## 5. The Natriuretic Peptide System

All that is known to date about NPs makes it possible, on careful reflection of the data, to believe that they represent an integrated system and do not constitute separate individual entities, each with its role (Figure 3). Supporting this assumption, first of all, is the structural similarity (Figure 1): each of the NPs are composed of a peptide ring that can maintain its circular structure thanks to a cysteine bridge, which is phylogenetically well-preserved in that it represents the region of the hormone that is capable of interacting with the receptor, thereby triggering downstream molecular pathways.

The presence of members of the NP family is reported in both the animal and plant worlds, and it appears that the most archaic member is the CNP, with a preserved structure [171]. ANP and BNP, on the other hand, seem to have been constituted by tandem duplications of the ancestral CNP gene, most likely in fish.

The fact that this happened, and that this hormonal system survived and was enhanced in phylogenesis, suggests its fundamental role in electrolyte and fluid homeostasis, the cardiovascular system, and other strategic targets [172].

Moreover, most members of this family are known to activate the same family of receptors with guanylyl cyclase activity, which can catalyze the cleavage reaction of guanosine triphosphate into cyclic guanosine monophosphate (cGMP) and pyrophosphate; cGMP, as a second messenger, mediates and regulates a variety of vital functions, from platelet aggregation to neurotransmission, from the genesis of libido to intestinal peristalsis, from blood pressure regulation to bone growth, and many others [173] (Figure 2).

As we have seen from the above, to carry out their functions, the NPs constitute a cross-talk between various systems, such as the endocrine system, the cardiovascular system, the renal excretory system, and the nervous system. Take, for example, the regulation of fluid and electrolyte balance. As a consequence of volume overload, there is the release of ANP and BNP due to the distension of the myocardial fibers [5] (Figure 3). These will maintain normal blood pressure levels by counteracting renin–angiotensin–aldosterone system (RAAS) activity, vasodilation, and stimulating renal diuresis [110].

Urodilatin, which we have seen as the real protagonist in maintaining the sodium balance, also intervenes in the kidney after the increase of renal blood perfusion pressure, which stimulates the excretion of excess sodium and water [42]. Moreover, urodilatin is also produced following atrial elongation and stimulation of carotid chemoreceptors, probably thanks to a neurological link between the heart and kidney, and between cephalic sodium chemoreceptors and the kidney, respectively [42,43]. Moreover, the sympathetic nervous system also intervenes at the renal level by stimulating the production of urodilatin [45]. After all, we have seen how NPs including ANP and BNP regulate the tone of the sympathetic autonomic nervous system in a negative direction, thus promoting a reduction in blood pressure. Previous studies have shown that microinjection of atrial natriuretic peptide into the caudal nucleus tractus solitarii produces significant increases in local neuronal firing rates associated with reductions in the arterial pressure in anesthetized Wistar rats. Single units excited by microinjections of atrial natriuretic peptide into the caudal nucleus tractus solitarii were also excited by the activation of arterial baroreceptors and were inhibited by baroreceptor unloading. To test the hypothesis that endogenous atrial natriuretic peptide in caudal nucleus tractus solitarii is involved in the tonic control of blood pressure in the rat, we administered a blocking monoclonal antibody to atrial natriuretic peptide in a volume of 50 nl artificial cerebrospinal fluid via microinjection into the caudal nucleus tractus solitarii of spontaneously hypertensive Wistar-Kyoto rats and observed the effects on mean arterial pressure and heart rate. Control injections of monoclonal antibody were administered into the rostral nucleus tractus solitarii, hypoglossal nucleus, spinal trigeminal nucleus, and cuneate nucleus of spontaneously hypertensive rats. Microinjection of monoclonal antibody into the caudal nucleus tractus solitarii were found to cause significant increases in mean arterial pressure in spontaneously hypertensive rats, but not in Wistar-Kyoto rats. There was no concomitant change in heart rate observed. Control injections of purified mouse immunoglobulin into the caudal nucleus tractus solitarii and of monoclonal antibody into the control neuronal groups listed above had no effect on mean arterial pressure. These results suggest that endogenous atrial natriuretic peptide in the caudal nucleus tractus solitarii mediates the tonic control of blood pressure in spontaneously hypertensive rats, but not in normotensive Wistar-Kyoto rats [142] (Figure 3). In addition, the effects of NPs at the brain level are complementary to those on other targets, reinforcing the latter: inhibition of salt appetite and stimulation of the sense of thirst stimulate natriuretic and diuretic actions at the kidney level [140] (Figure 3). Finally, we have described how GN and UGN can constitute an endocrine axis between the intestine and kidney that stimulates diuresis and natriuresis in response to a salty meal [47] (Figure 3). All these observations lead us to think that there is an extensive functional interconnection between the various members of the natriuretic peptide family. Therefore, it is likely to think that they are not separate entities but are a real system of functionally interconnected NPs. Observing the NP family from this perspective could allow us to better understand these molecules’ behavior and function, opening up new and exciting scenarios in understanding these numerous physiological and pathological processes.

An example of what has just been said could concern hyponatremia. There is a growing awareness that mild chronic hyponatremia is associated with a mental dysfunction, unsteady gait, osteoporosis, increased falls, and bone fractures [174,175]. In addition, several authors have investigated the direct effects of chronic hyponatremia on bone and brain metabolism [176,177]. Nevertheless, we can speculate that the neurological and bone changes observed in patients with RSW and chronic hyponatremia may be mediated not only by the hyponatremia itself, but by an “impairment” of the NP system, which, as we have seen, regulates the metabolism of the nervous and bone systems, in addition to the electrolyte balance [19].

The enthusiasm for scientific research toward NPs has grown exponentially over the past decades, and the clinical and experimental trials have made it possible to carry out numerous steps going forward, understanding that in addition to their known cardiovascular effects, NPs represent an integrated system of hormones with the endocrine role, and encompass nervous system and immune system regulatory functions. It seems clear that some aspects still need to be clarified, and that the road to their use for therapeutic purposes has not been fully mapped out, but further understanding of what is not yet known will certainly help to imagine a future and imminent use of these agents in the treatment of several diseases.

## Data Availability

No new data were created or analyzed in this study. Data sharing is not applicable to this article.
